# A cluster of Zika virus infection in a Chinese tour group returning from Fiji and Samoa

**DOI:** 10.1038/srep43527

**Published:** 2017-03-07

**Authors:** Jimin Sun, Tao Fu, Haiyan Mao, Zhen Wang, Junhang Pan, Shannon Rutherford, Jiangping Ren, Xuanjun Dong, Yin Chen, Zhihong Zhu, Xiaohua Qi, Zhenyu Gong, Qiyong Liu, Hongjie Yu, Liebo Zhu, Wenxian Chen, Zhiping Chen, Yanjun Zhang, Enfu Chen

**Affiliations:** 1 Zhejiang Provincial Center for Disease Control and Prevention, Hangzhou, China; 2 State Key Laboratory for Infectious Disease Prevention and Control, Collaborative Innovation Center for Diagnosis and Treatment of Infectious Diseases, National Institute for Communicable Disease Control and Prevention, Chinese Center for Disease Control and Prevention, Beijing 102206, China; 3 Yiwu Municipal Center for Disease Control and Prevention, Yiwu, China; 4 Centre for Environment and Population Health, School of Environment, Griffith University, Nathan QLD 4111, Australia; 5 Division of Infectious Disease, Key Laboratory of Surveillance and Early-warning on Infectious Disease, Chinese Center for Disease Control and Prevention, Beijing, China

## Abstract

Zika virus is currently causing extensive outbreaks in a number of countries in South and Central America and the Caribbean and has been associated with foetal abnormalities. We report an outbreak of Zika virus infection in a Chinese tour-group returning from a nine day holiday in Fiji and Samoa. The index case was a 38-year old male who developed symptoms while travelling back from Fiji to Hong Kong on the 14th February, 2016. A field investigation was initiated to define the epidemiological, clinical and virological characteristics of Zika virus infection in this tour group and revealed two further symptomatic infections and one asymptomatic infection among the 33 travellers; an overall infection attack rate of 12% in these travellers. Active surveillance led to detection of Zika virus RNA in the serum of one case four days prior to onset of symptoms and detection of Zika virus in saliva from one asymptomatic infection.

Zika virus, related to dengue, yellow fever, Japanese encephalitis, and West Nile viruses, is an arbovirus belonging to the family *Flaviviridae* and the genus *Flavivirus*^1^,^2^. The virus was isolated in 1947 from a rhesus monkey in the Zika forest near Entebbe, Uganda^3^. It is believed to be transmitted to humans by daytime-active *Aedes* mosquitoes, such as *Aedes aegypti, Aedes albopictus, Aedes africanus*, and *Aedes luteocephalus*^4^,^5^,^6^,^7^. The clinical presentations of Zika virus infections include self-limiting acute febrile illnesses with fever, headache, myalgia, retro-orbital pain, arthralgia, conjunctivitis, and rash which are closely resemble those that are caused by dengue virus and Chikungunya virus. Although serologic evidence indicated Zika virus might have been epidemic in some countries of Africa and Asia, no outbreaks and only 14 cases of human Zika virus disease have been previously documented until 2007^8^,^9^,^10^. An outbreak of Zika fever took place in Yap Island in 2007 and a large epidemic was reported in French Polynesia in 2013^11^,^12^. In late 2014, Brazil detected a cluster of cases of febrile rash in the northeast region of the country and Zika virus was confirmed as the cause of the cluster in May 2015. Shortly afterwards, the virus spread quickly in South and Central America, and the Caribbean. As of July 27th 2016, 67 countries and territories reported continuing mosquito-borne Zika virus transmission. Of these countries and territories, 50 countries are experiencing the first outbreak of Zika virus since 2015, with no previous evidence of circulation, and with ongoing transmission by mosquitos^13^.

In mainland China, the first imported Zika case was confirmed on February 9^th^ 2016, and a total of 9 cases were reported from February 1^st^ to 29^th^, 2016^14^. Among these, one Zika case who had visit history to Fiji and Samoa was identified in Zhejiang Province, China. Besides the investigation of the index case, 32 individuals in the travel group were also investigated and consented to medical observation. Subsequently, two Zika cases and one asymptomatic infection were identified. This is the first cluster of Zika virus infection in a Chinese tour group who had been visiting an area of active transmission. Here, we report characteristics of the cluster; information valuable for the control and prevention of Zika virus in nonendemic areas.

## Results

The travel group comprised 33 persons, 17 male and 16 female. The median age of participants was 37 years (range, 3–75 years). Serum, saliva, and urine of the index patient were collected on February 16^th^ and similar samples for the other 32 participants were collected on February 17^th^. Except the serum sample of a three year old boy which was not available, 32 serum, 33 saliva and 33 urine samples were collected from the travel group. Besides the index patient, samples from three people in the group were positive for Zika virus. Serum, saliva, and urine were collected daily when any sample for that person was found positive for Zika virus. Furthermore, saliva and urine were collected from the other participants on February 19^th^, February 22^nd^, and February 25^th^.

A total of four Zika virus infections including three patients and one asymptomatic infection were identified. The index case was a 38-year old male. He and the other 32 persons (his family members and friends) travelled to the republic of Fiji (Fiji) by air on February 4^th^, 2016. They reached Fiji at 7:10 am, February 5^th^ and went sightseeing until February 7^th^. They reported that they mainly swam and rowed a boat. Although they did not have a history of mosquito bites in Fiji, they wore short-sleeved shirts and short pants and the mosquito density was very high.

**Figure 1:**
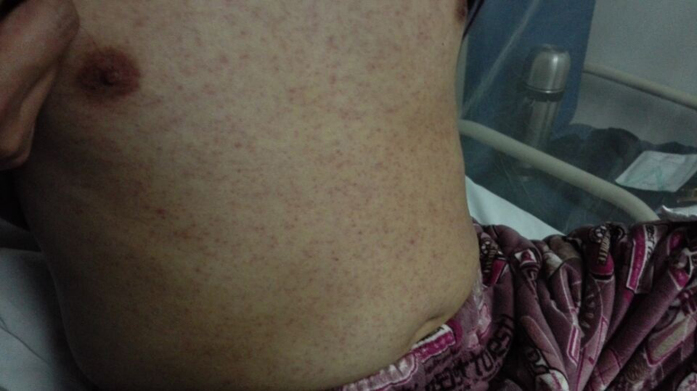
Rashes on the body of the index cases on February 17^th^, 2016.

**Figure 2:**
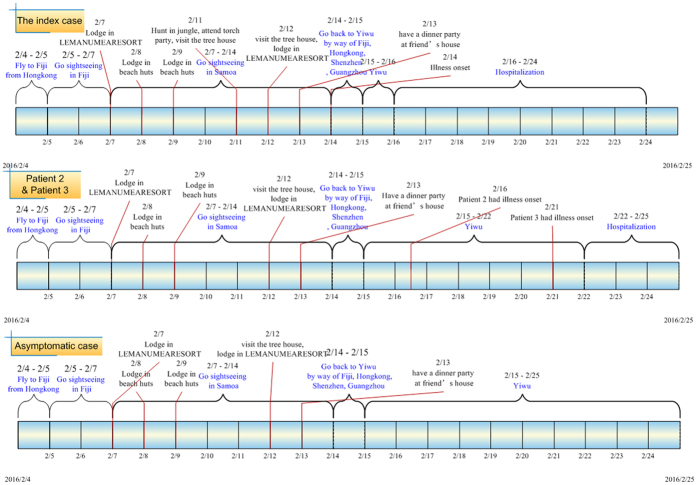
Timelines of events associated with the cluster of Zika virus infection in China.

They left Fiji at 14:00 and arrived in the independent state of Samoa (Samoa) at 18:50, February 7^th^. During the period in Samoa, the index case swam, rowed a boat, hunted in the jungle, attended a torch party (a nighttime cultural) and visited tree houses. The index case reported a history of mosquito bites during the latter three activities. The group returned to Yiwu, China by way of Fiji, Hong Kong, Shenzhen and Guangzhou from February 14^th^ to 15^th^. On February 14^th^, the index case developed fever, chills and diarrhea when he was in the airplane from Fiji to Hong Kong and his body temperature was 38.5 °C at 16:48 on February 14^th^. He had rashes behind his ears on the evening of February 15^th^ and the rashes expanded to his body (February 16^th^, Fig. 1) and legs (February 17^th^). The rashes began to subside from February 18^th^ and disappeared on February 20^th^. His body temperature was 37.3 °C at 16:00 on February 16^th^ and returned to normal from February 17^th^. Saliva and urine tested positive for Zika virus by Zhejiang Provincial Center for Disease Control and Prevention (ZJCDC) on the evening of February 16^th^. Then samples were transported to the National Institute for Viral Disease Control and Prevention, Chinese Center for Disease Control and Prevention (China CDC) on February 17^th^ and were confirmed to be positive for Zika virus on February 18^th^. The index case was hospitalized from February 16^th^ to February 24^th^ (Fig. 2). No Zika virus RNA was found in serum and saliva of the index patient from February 17^th^. However, Zika virus RNA was found in urine from February 16^th^ to February 20^th^ (Table 1).

Case 2, case 3 and the asymptomatic infection were family members. Case 2 was the son, case 3 was the father and the asymptomatic infection was the mother. Case 2 was an 8-year old boy, case 3 was a 36-year old male, and the asymptomatic infection was a 34-year old female. These three cases were in the same travel group (Fig. 2). Notably, they remembered that the mosquito density was very high and they had been bitten by mosquitoes in Samoa. Case 2 developed a headache on February 16^th^ and rashes appeared on his back (February 21^st^) and face (February 22^nd^). The rashes began to subside on February 23^rd^ and disappeared on February 25^th^. Zika virus RNA was found in his serum (from February 17^th^), saliva (from February 19^th^) and urine (from February 22^nd^).

Case 3 developed fever (37.4 °C), rashes and conjunctivitis on February 21^st^. However, Zika virus RNA was found in his serum sample collected on February 17^th^. Serum, saliva and urine of case 3 which were collected on February 19^th^ were all positive for Zika virus. Serum and saliva samples collected on February 20^th^ were also positive (Table 1). There were obvious rashes on his body on February 22^nd^ subsiding gradually from February 23^rd^ and disappearing by February 25^th^.

The asymptomatic infection was the wife of case 3, and she reported that she hadn’t had sexual contact with case 3 from February 4^th^ to February 18^th^. Saliva collected on February 19^th^ was positive for Zika virus (Table 1).

**Figure 3:**
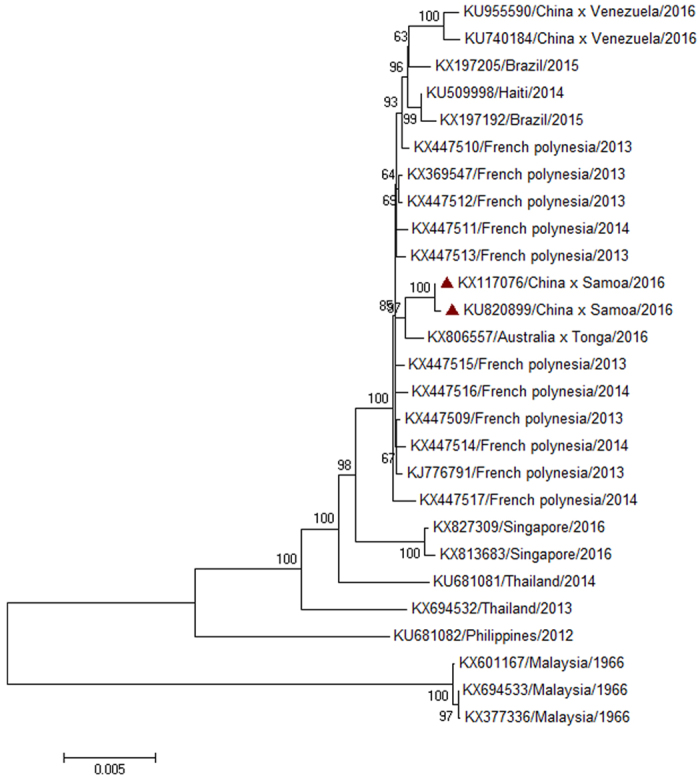
Molecular Phylogenetic analysis of complete genes of Zika virus isolated from Zhejiang Province, China.

We submitted sequences of the index patient (KU820899) and case 2 (KX117076) to GenBank on March 7^th^, and April 22^nd^, 2016, respectively. Our sequences were identical to corresponding parts of other three sequences (KU955589, KX253996, KU866423) in GenBank. According to published papers^15^,^16^,^17^, these three sequences were from the same case in our study (the index patient) and these sequences were submitted to GenBank on April 11^th^ (KU955589), May 18^th^ (KX253996), June 13^th^ (KU866423), 2016, respectively. Phylogenetic analysis showed that complete sequences of Zika virus in our study belong to the same clade (Fig. 3). They were also most similar to the sequence (KX806557) which was from Tonga and some sequences from French polynesia. But the two sequences in our study didn’t belong to the same clade with another two sequences (KU955590, KU740184) of cases in mainland China who were from Venezuela.

## Discussion

Since early 2015, a widespread outbreak of Zika fever, caused by the Zika virus, is ongoing, primarily in the Americas. The outbreak began in April 2015 in Brazil, and has spread to other countries in South America, Central America, Mexico, and the Caribbean. Travel-related imported infections have also been increasingly reported in many countries. In China, the first Zika case from Samoa and Fiji was identified by the General Administration of Quality Supervision, Inspection and Quarantine of the People’s Republic of China (AQSIQ)^14^. Then two other patients and one asymptomatic infection were identified in the same travel group under the collaboration of AQSIO and ZJCDC. However, the real number of individuals infected by Zika virus in the travel group may be larger. As Zika is emerging in China and we haven’t yet establish methods for detection of IgM or IgG antibodies for the virus, we do not know whether other individuals whose serum, saliva and urine were negative for Zika virus RNA were infected with Zika virus. The observed infection attack rate in this tour group was 12%. However, this is likely to be a gross under-estimate given previous estimates that 80% of Zika infections are asymptomatic.

Due to the fact that most Zika virus infections are asymptomatic and symptoms of Zika fever are non-specific, it is impossible to identify all imported Zika virus infections in China. Till now, Zika virus is reported to be pandemic in only some areas including South America, Central America, and the Caribbean. Moreover, the amount of travel between China and Zika virus endemic areas has increased after Spring Festival. It is inevitable that more and more Zika virus infections will be imported to China and most of them can’t be identified. Furthermore, *Aedes* mosquitoes, vectors for Zika virus, are found across many provinces of China^18^,^19^,^20^. Outbreaks of dengue fever which is transmitted by the same *Aedes* mosquitoes have occurred frequently in China in recent years^21^,^22^,^23^. Similarly, it is likely that local transmission and outbreaks of Zika virus disease will take place frequently in the future in China based on an increasing number of imported Zika virus infections, the difficulties in identification of all these Zika virus infections, the wide distribution and high density of mosquitoes capable of transmitting Zika virus and susceptibility of Chinese individuals.

Case 3 developed symptoms of fever on February 21^st^, but his serum had been positive for Zika virus since February 17^th^. One study reported that 42 (3%) of 1505 blood donors were found positive for ZIKAV by PCR although they were asymptomatic at the time of blood donation in French Polynesia^24^. Of the 42 donors tested positive by RT-PCR, 11 declared that they had a Zika fever-like syndrome from 3 to 10 days after they gave blood^24^. Our findings further suggest that cases might be infectious during the incubation period. As no symptoms develop during the incubation period, it is very hard to identify cases who have been infected hence these individuals can be a source of infection and help Zika virus circulation.

An asymptomatic infection was also identified in our study. Although it is possible that this is a false positive or a result of cross contamination as only the patient’s saliva sample was positive for Zika virus, we can’t rule out that Zika virus could have been detected in serum or urine if these samples had been collected daily. Consequently, we suggest that persons who come from Zika virus endemic areas should reduce their sexual activities and outdoor activities during mosquito active period for 12 days even if they don’t develop symptoms of Zika fever.

In our study, Zika virus RNA was found in serum, and also in saliva and urine. Similar to dengue virus and West Nile virus^25^,^26^, Zika virus can be detected in urine for a longer time than in serum. These results were similar to the results of another three studies^27^,^28^,^29^. In order to improve the diagnosis sensitivity of Zika virus infections, we suggest that serum, saliva and urine should be collected simultaneously. As of February 2016, there have been three reported cases of sexual transmission, with at least 14 additional reported cases of possible sexual transmission under investigation^29^,^30^,^31^,^32^. As Zika virus exists in saliva or urine for a longer time, we speculate that exchange of saliva and contact with residual urine during sexual intercourse may also transmit Zika virus. It is interesting that both persons from whom virus was detected in the urine were male, although one was only 8 years old.

Consistent with other studies, symptoms of Zika fever were milder than dengue fever and chikungunya fever^33^. The symptoms reported in this investigation were low grade fever, rash, conjunctivitis and headache lasting not more than 7 days. Complete blood counts (white blood cell, platelet, etc.) of three patients were normal also indicating that symptoms of Zika fever were not serious in these patients.

There are several limitations of our study. First, serum was not collected daily from the other persons in the travel group. Second, semen of all adult males in the group were not available. Third, detection methods of Zika virus IgM and IgG had not been established in our laboratory. As a result, we don’t know whether other participants have been infected with Zika virus.

In summary, we identified the first cluster of Zika virus infections in China among a group who travelled to Fiji and Samoa. It is likely that the outbreak of Zika virus infection in China might occur in *Aedes* mosquito active seasons triggered by imported cases. Our study also indicates cases during the incubation period and asymptomatic infections might be infectious. They might play an important role in the transmission of Zika virus even when they have no symptoms. These results indicate that more research should be done to explore the role of Zika cases during the incubation period and asymptomatic infections in the spread and transmission of Zika virus.

## Methods

### Case definition

In accordance with the document entitled “The diagnosis and treatment programs of Zika virus disease” issued by the National Health and Family Planning Commission of the People’s Republic of China^34^, a person with symptoms (e.g., fever, rash, arthralgia or conjunctivitis) without obvious alternative causes and with epidemiological risk factors (a history of travel to regions where Zika virus is endemic within 14 days of illness onset) is defined as a suspected case of Zika virus disease. Confirmed cases of Zika virus infection were defined as those who met the criteria for a suspected case and who also met one or more of the following criteria: (1) detection of Zika virus RNA by a molecular method, (2) isolation of Zika virus.

### Epidemiological investigation and medical observation

On February 16^th^, 2016, Guangdong Provincial Center for Disease Control and Prevention (CDC) informed ZJCDC that they found a suspected Zika case who had entered China through a port in Guangdong Province and then went to Yiwu City, Zhejiang Province, China. Epidemiologists from ZJCDC and Yiwu Municipal CDC (YWCDC) conducted field investigation of the index case and all persons in the travel group. A standardized questionnaire was used to collect demographic information such as age, gender, occupation, residential address and exposure history. All individuals in the travel group were placed under daily active surveillance for fever, rash, arthralgia or conjunctivitis for 12 days following their departure from Samoa. Individuals were taken to hospital as soon as possible when they developed symptoms such as fever, rash, arthralgia or conjunctivitis.

### Ethics statement

The research protocol was approved by the ethics committee of Zhejiang Provincial Center for Diseases Control and Prevention. All participants provided written informed consent. For children, parents or guardians of eligible children were informed and asked to provide written informed consent on behalf of their children. The aims of our study were explained to all of them, and their written consent was obtained before inclusion onto this study.

### Samples collection and virus detection

Samples including serum, saliva and urine of the index case and two other cases were collected daily once the case was hospitalized. In order to identify whether individuals who had the same exposure history were infected with Zika virus, samples including serum, saliva and urine were also collected once from participants on February 17^th^, 2016. The viral RNA of each sample was extracted using the QIAamp viral RNA minikit according to the manufacturer’s instructions (QIAGE, Hilden, Germany). Then the viral RNA was screened with a rRT-PCR assay targeting the Zika virus gene with the primers ZIKVF 9121–9141 (5′-CCT TGG ATT CTT GAA CGA GGA-3′) and ZIKVR 9312–9290 (5′-AGA GCT TCA TTC TCC AGA TCA A-3′). The temperatures and cycling times are as follows: 50 °C 10 minutes, 1 cycle; 95 °C 30 minutes 1 cycle; 95 °C 15 seconds and 55 °C 45 seconds, 40 cycles. All experiments were performed in accordance with relevant guidelines and regulations. The assay was provided by the National Institute for Viral Disease Control and Prevention, China CDC and the institute approved the experiments.

### Genome sequencing and phylogenetic analysis

Viral RNA was extracted from the samples of Zika patients using the QIAamp viral RNA minikit (Qiagen, Hilden, Germany). Samples were sequenced based on the Ion torrent PGM platform. Sequencing data were analyzed with Geneious v. 8.1.7. Complete genomes for the index patient and case 2 were submitted to GenBank (accession number: KU820899, KX117076). We searched all available Zika full genomes from Asia and the South Pacific as of October 1^st^, 2016. The Maximum likelihood (ML) phylogenetic trees model was used to generate the phylogenetic trees of complete sequences of Zika virus. We used the General Time Reversible Nucleotide substitution model and Gamma distributed with Invariant site (G + I) rates among sites with a bootstrapping resampling process (500 replications) implemented in MEGA version 7.

## Additional Information

**How to cite this article**: Sun, J. *et al*. A cluster of Zika virus infection in a Chinese tour group returning from Fiji and Samoa. *Sci. Rep.* **7**, 43527; doi: 10.1038/srep43527 (2017).

**Publisher's note:** Springer Nature remains neutral with regard to jurisdictional claims in published maps and institutional affiliations.

## Figures and Tables

**Table 1:** RT-PCR results for serum, saliva and urine collected on different time from Zika virus cases and the asymptomatic infection.

		The index case	Case 2	Case 3	The asymptomatic infection
February 14^th^	Serum	+	NA	NA	NA
	Urine	NA	NA	NA	NA
	Saliva	NA	NA	NA	NA
February 15^th^	Serum	NA	NA	NA	NA
	Urine	NA	NA	NA	NA
	Saliva	NA	NA	NA	NA
February 16^th^	Serum	−	NA	NA	NA
	Urine	+	NA	NA	NA
	Saliva	+	NA	NA	NA
February 17^th^	Serum	−	+	+	−
	Urine	−	−	−	−
	Saliva	−	−	−	−
February 18^th^	Serum	−	NA	NA	NA
	Urine	−	NA	NA	NA
	Saliva	−	NA	NA	NA
February 19^th^	Serum	−	+	+	−
	Urine	+	−	+	−
	Saliva	−	+	+	+
February 20^th^	Serum	−	NA	+	NA
	Urine	+	−	−	−
	Saliva	−	+	+	−
February 21^st^	Serum	−	NA	+	NA
	Urine	−	−	−	−
	Saliva	−	+	+	−
February 22^nd^	Serum	−	+	NA	−
	Urine	−	+	+	−
	Saliva	−	+	+	−
February 23^rd^	Serum	−	NA	NA	NA
	Urine	−	−	+	−
	Saliva	−	+	+	−
February 24^th^	Serum	−	NA	NA	NA
	Urine	−	+	+	−
	Saliva	−	−	−	−
February 25^th^	Serum	NA	NA	NA	NA
	Urine	−	+	+	−
	Saliva	−	−	−	−

^*^+, positive; −, negative; NA, not available.
